# The Role of Dendritic Cells in Fibrosis Progression in Nonalcoholic Fatty Liver Disease

**DOI:** 10.1155/2015/768071

**Published:** 2015-08-03

**Authors:** Paloma Almeda-Valdes, Nancy E. Aguilar Olivos, Beatriz Barranco-Fragoso, Misael Uribe, Nahum Méndez-Sánchez

**Affiliations:** ^1^Endocrinology and Metabolism Department, Instituto Nacional de Ciencias Médicas y Nutrición Salvador Zubirán, 14080 Mexico, DF, Mexico; ^2^Liver Research Unit, Medica Sur Clinic & Foundation, 14050 Mexico, DF, Mexico; ^3^Department of Gastroenterology, National Medical Center “20 Noviembre”, 03229 Mexico, DF, Mexico

## Abstract

Nonalcoholic fatty liver disease (NAFLD) is the most frequent cause of chronic liver disease. NAFLD encompasses a wide range of pathologies, from simple steatosis to steatosis with inflammation to fibrosis. The pathogenesis of NAFLD progression has not been completely elucidated, and different liver cells could be implicated. This review focuses on the current evidence of the role of liver dendritic cells (DCs) in the progression from NAFLD to fibrosis. Liver DCs are a heterogeneous population of hepatic antigen-presenting cells; their main function is to induce T-cell mediated immunity by antigen processing and presentation to T cells. During the steady state liver DCs are immature and tolerogenic. However, in an environment of chronic inflammation, DCs are transformed to potent inducers of immune responses. There is evidence about the role of DC in liver fibrosis, but it is not clearly understood. Interestingly, there might be a link between lipid metabolism and DC function, suggesting that immunogenic DCs are associated with liver lipid storage, representing a possible pathophysiological mechanism in NAFLD development. A better understanding of the interaction between inflammatory pathways and the different cell types and the effect on the progression of NAFLD is of great relevance.

## 1. Introduction

**Figure 1:**
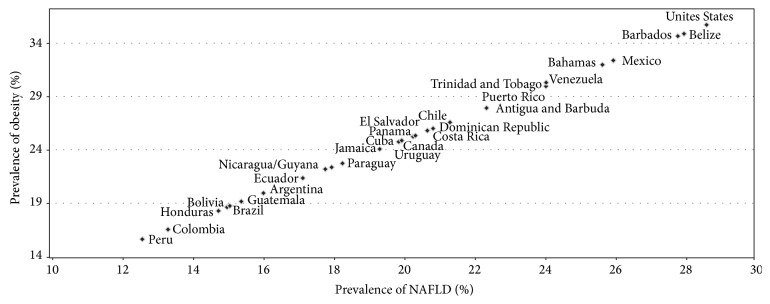
Correlation between the prevalence of obesity and NAFLD in the Americas. The graph was built with data from the prevalence of obesity for each country; NAFLD prevalence was estimated assuming that about 80% of obese patients might develop NAFLD in the Americas countries. Reproduced with permission of the journal [1].

Nonalcoholic fatty liver disease (NAFLD) is the most frequent cause of liver disease in the Western world. Its prevalence among liver diseases, and in the general population, has been rising in recent years along with associated conditions, including obesity, insulin resistance, metabolic syndrome, and diabetes. Interestingly, in a recent study, we analyzed the epidemiology of NAFLD in the Americas based on the prevalence of obesity, and we found that the estimated prevalence rates of NAFLD were higher in the United States (29%) and in Mexico (26%), countries that also have a high prevalence of obesity. The prevalence of NAFLD and obesity is directly correlated (Figure 1) [1]. In addition, the prevalence of NAFLD in Europe and the Middle East ranges from 20% to 30% [2–5], whereas the prevalence of NAFLD in Japan and China is similar to that in Europe [6].

**Figure 2:**
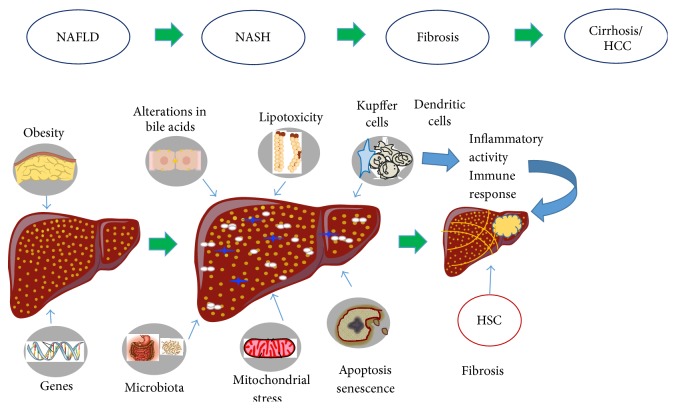
Schematic mechanisms involved in the progression of NAFLD to NASH. Lifestyle factors such as obesity and genetic predispositions contributing to the development of insulin resistance and hepatic steatosis. In the following steps multiple parallel metabolic hits lead to cellular damage, via a process called “lipotoxicity.” Injured hepatocytes initiate an inflammatory response, predominantly via toll-like receptors, and activate proinflammatory signaling pathways in the setting of increased adipokine levels. Also the apoptosis and senescence are alternative cell fates that are likely to be of greater importance to disease progression. Direct recruitment of Kupffer cells and other components of the innate immune response such as dendritic cells occurs with activation of the inflammasome and the coordinated release of proinflammatory and profibrogenic cytokines and ligands. Hepatic stellate cells (HSCs) are subsequently activated to produce extracellular matrix leading to progressive fibrosis and cirrhosis and its complications such as hepatocellular carcinoma (HCC).

NAFLD is characterized by fat deposition in hepatocytes, mainly in the form of triglycerides. In most individuals, hepatic steatosis is present as a benign condition. However, a proportion of patients with NAFLD develops inflammation and necrosis with or without fibrosis and evolves to nonalcoholic steatohepatitis (NASH). The pathogenesis of NASH has not been completely elucidated; multiple inflammatory and noninflammatory factors are implicated. The hepatic lipid deposition induces oxidative stress and hepatic cell injury, with subsequent inflammatory cell infiltration. Lipid accumulation triggers proinflammatory cytokines (CTN) linked to the activation of hepatic cellular subtypes that sustain CTN production (Figure 2) [7]. Interestingly, in recent years, it has been reported that liver dendritic cells (DCs) appear to be involved in liver fibrosis in NAFLD [8], although the role of DC in liver disease has not been clearly defined. This review focuses on the current evidence of the role of DC in fibrosis progression in NAFLD.

## 2. Liver Dendritic Cells

**Figure 3:**
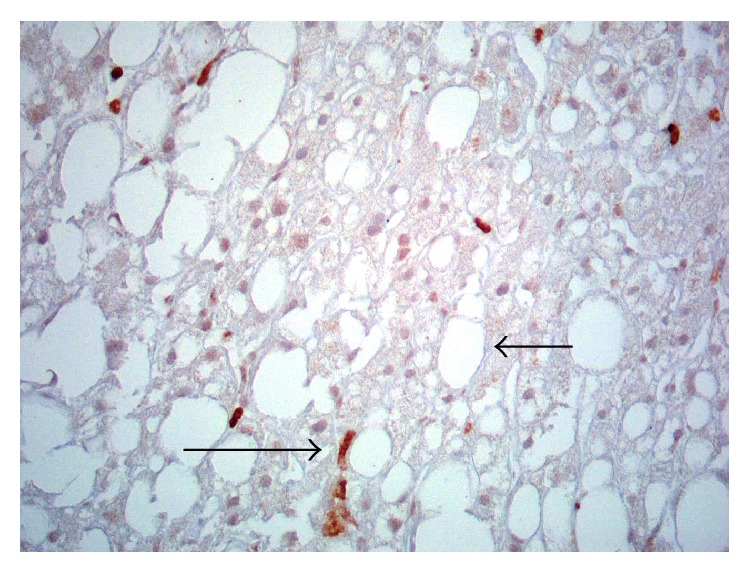
Immunohistochemistry for hepatic dendritic cells. Some dendritic cells are CD11C positive (large arrow) in a patient with nonalcoholic fatty liver disease. The vast majority of hepatocytes contained vacuoles of lipids (small arrow) (grade 3 micro- and macrovesicular steatosis) (×400).

Liver DCs are a heterogeneous population of hepatic sinusoidal antigen-presenting cells, found preferentially in the periportal and pericentral space and constituting less than 1% of the nonparenchymal cells [9]. They are part of the hepatic reticuloendothelial system, which also includes sinusoidal endothelial cells and Kupffer cells [10]. In particular, DCs have a migratory capacity and a remarkable ability to produce CTN; this feature distinguishes liver DC from Kupffer cells and promotes the adaptive immune system response [11]. Liver DC can be defined in broad terms, such as CD45^+^ cells with a high expression of major histocompatibility complex class II (MHCII) and the absence of other hematopoietic markers [11], but many markers are needed to identify dendritic cell subsets [12]. In murine models, three subsets of hepatic DC (CD19−, CD11c+) have been characterized: lymphoid (CD8*α*+, B220−, and CD11b−), myeloid (CD8*α*−, B220−, and CD11b+), and plasmacytoid (B220+, CD11b−). The first two subtypes are denoted as conventional DC and are located at the periportal region and central veins whereas the plasmacytoid DC subtypes are located in the liver parenchyma. Another liver DC subtype containing mixed features of the myeloid and lymphoid subtypes has been characterized [13]. In addition, a new subset called natural killer dendritic cell which has an intermediate development state has been recognized [14]. On Table 1 the different liver DC subsets with its markers are shown.  Figure 3 shows a hepatic DC displaying positivity for CD11c.

Natural killer T cells (NKT) are a group of T lymphocytes that express both NK, such as CD161 and CD94, and T-cell markers. These cells comprise 20 to 35% of mouse liver lymphocytes and 10 to 10% of rat and human liver lymphocytes. NKT recognize lipid antigens and respond to injury stimulating Kupffer cells, hepatocytes, and DC [15]. In animal [16] and human [17] NAFLD models, an inverse correlation between NKT cells and accumulation of hepatic lipid has been reported. NKT cells generate both Th1 and Th2 cytokines; therefore, NKT depletion may result in an increase of Th1 cytokines such TNF-*α*, IL-2, and IFN-*γ* [18]. In contrast, some other studies have shown NKT cells accumulation as NAFLD progresses [19]. Liver DCs stimulate the release of proinflammatory CTN release by NKT cells and become activated after the NKT cells population is eliminated [20].

Because of the heterogeneity of DC and our limited understanding of their role in human tissues, the contribution of these cells to the development of fibrosis is unclear at this point [21].

## 3. Liver Dendritic Cells in Health and Disease

DCs are derived from hematopoietic progenitor cells in the bone marrow.* In vitro* and* in vivo* studies have shown that DCs are potent inducers of immune responses. Their main function is to induce T-cell mediated immunity by antigen processing and presentation to T cells. The features of DC include their ability to capture antigens, the capability of processing and presenting peptides, and migration to lymphoid organs [22]. On the other hand, DC can prevent activation of T cells, playing an important role in preventing autoimmunity and rejection [23].

DCs process and present antigens to T lymphocytes. Fragments of antigens bound to major histocompatibility complex (MHC) molecules are recognized by receptors in the T cells. MHC class I molecules stimulate cytotoxic T cells, and MHCII molecules stimulate T helper cells. Activation of cytotoxic T cells can kill target cells, whereas activation of T helper cells can induce regulatory immune properties. The induction of a T-cell immune response requires peptide recognition by T cells. DCs bring antigens to T cells and express costimulatory molecules, facilitating the induction of the immune response [22].

DCs are present in the skin [24], inner lining of the airways [25], interstitial spaces of diverse organs [26], lymphoid tissues [27], and blood [28]. In humans, after contact with antigens, DCs migrate from the peripheral tissues to the lymphoid organs [29]. Self-antigens captured and presented by DC may induce tolerance [30]. DCs are capable of maintaining tolerance and inducing immunity in different contexts (steady state versus inflammation) [31]. Furthermore, it has been suggested that the role of DC is mainly determined by their state of development. DCs exist in two conditions: immature and mature. Most DCs in the peripheral tissues are in the immature state.

*In vitro*, immature dendritic cells (IDC) capture antigens, phagocyte particles [32], form pinocytic vesicles, and express receptors to mediate endocytosis, including C-type lectin receptors (such as the macrophage mannose receptor) and DEC-205, as well as Fc*γ* and Fc*ε* receptors [33]. IDC in mice possess compartments rich in MHCII, where MHCII-peptide complexes are formed [34]. IDC may prompt tolerance by diverse mechanisms such as deletion of T cells and induction of regulatory T cells. In this state, they express low levels of surface MHC class I and II and costimulatory molecules. They capture self-antigens and innocuous environmental proteins and target them to MHCII in the lysosomes, but they are not used for the formation of MHCII-peptide complexes [35]. IDC in lymphoid tissues perform endocytosis and express low levels of costimulatory molecules such as CD86 and CD40. The ability of IDC to form MHCII-peptide complexes may be important to tolerate T cells [23]. In the thymus, DCs delete self-reactive T-cell clones [36]. In animal models, IDC circulate through tissues, enter afferent lymphatics, and migrate to the T-cell area, where they die [28]. In this way, DCs play a role in developing immune tolerance: presenting self-antigens to T cells, deleting autoreactive lymphocytes, and inducing regulatory T-cell formation, which induces tolerance by suppressing the responses of T cells to stimuli [23].

When DCs are exposed to immune or inflammatory signals, including microbial products and proinflammatory CTN, they undergo maturation and they are directed to the T-cell areas of the lymphoid organs, such as the spleen and lymph nodes. CTN including IL-1, GM-CSF, and TNF-*α* stimulate human DC maturation, whereas IL-10 blocks it [37]. In addition, DC migration involves adhesion molecules and chemotactic factors [38]. Mature dendritic cells (MDC) have a reduced capacity to take up antigens but can participate in the immune response by stimulating T cells. They process antigens and present them bound to MHC molecules, initiating immunity [36]. Maturation causes changes in the phenotype of human DC. These changes include an increase in the production of MHCII-peptide complexes [35], expression of costimulatory molecules with the ability to bind T cells, and the production of CTN [39].

The changes in DC associated with maturation that makes them immunogenic include the redistribution of MHCII molecules from the intracellular compartments to the plasma membrane [40] and the expression of molecules that interact with receptors in T cells to increase adhesion and signaling. In mice identified molecules include liver fibrosis A-3/CD58, ICAM-1/CD54, and B7-2/CD86 [41]. In addition, human MDC synthetize CTN, chemokines (CHM), and receptors that promote their movement to lymphoid organs [42, 43].

The traffic of DC is a critical step for their immune function.* In vitro* studies have demonstrated that CHM are major regulators of DC migration. Both immature and MDC produce CHM, constitutively and by stimulation, respectively [44]. Inductors of the maturation of DC also change their migratory characteristics. Inflammatory CHM may function as signals for the localization of DC in nonlymphoid organs. This change may have a role in allowing DC to leave peripheral tissues. There is a large heterogeneity among DC in expression and responsiveness to CHM, modulated by their maturation.

CCR1, CCR2, and CCR5 are chemokine receptors responsible for migration of mice IDC to areas of inflammation [9]. In addition, maturation of DC induces a change in the expression of CHM receptors such as CCR7 and increases the levels of the platelet-activating factor (PAF) receptor. MDC are characterized by a high expression of CXCR4 and low levels of CCR4. These changes in the expression of CHM receptors and other ligands appear to be an important mechanism for localization and/or migration of DC in response to stimuli [43].

The increase in surface MHCII molecules is a hallmark of the maturation of DC.* In vitro* studies have shown that in IDC MHCII molecules are collected in endosomes and lysosomes, whereas, in MDC, MHCII molecules gather at the cell surface [45]. Dendritic cell lysosomes can confiscate antigens for long periods and form MHCII-peptide complexes [40]. Lysosomes may provide a mechanism for the regulation of the T-cell receptor ligands [23].

DCs mature by exposure to microbial or viral pathogens. In mice, microbial products enhance the production of CTN by DC, such as IL-12 [46]. Inflammatory CTN stimulate innate (natural killer cells) and acquired (B and T cells) immunity [22]. The toll-like receptor (TLR) and tumor necrosis factor receptors, mainly CD40, have a prominent role in the maturation of DC [31].

In humans, MDC initiate T-cell responses and can also differentiate the immune response to Th1 or Th2 types [47]. DC can affect the immune response in different ways according to the type of T cell that they stimulate. In mice, DC can activate cytotoxic T cells that interact with MHC class I-expressing cells. They can also activate T helper cells that can turn into Th1 cells in the presence of DC and IL-12. Th1-helper cells produce interferon-*γ* (IFN-*γ*) that activates macrophages. Macrophages promote differentiation of T cells into killer cells. On the other hand, IL-4 DCs induce T cells to differentiate into Th2 cells, which secrete IL-5 and IL-4. These CTN activate eosinophils and help B cells to produce antibodies [46].

The maturation of DC can occur not only in response to infectious agents but also during transplantation [48], contact allergy [49], and autoimmunity. In these circumstances, the necessary receptors for maturation have yet to be identified. TNF family members on mast cells and platelets and members of the hematopoietin family (such as granulocyte macrophage colony-stimulating factor, IL-4, and IL-13) influence DC development and maturation. These alternative incentives may produce DC with different functions or may be required in concert with TLR signaling for complete dendritic cell activity. In addition, different stimuli may produce different states of maturation [23].

DCs appear to also induce central and peripheral tolerance to autoantigens. In the thymus, T-cell deletion occurs, and, in lymphoid organs, this is caused by the induction of anergy or deletion of mature T cells. On the periphery, DC can capture and present autoantigens to T cells and induce tolerance to proteins that have no access to the thymus [22].

DCs play a role in diseases including infections, tumors, and infection with human immunodeficiency virus (HIV). MDC help in diffusing human HIV to T cells. Some tumors do not provoke a specific T-cell response; this may be because of the absence of functional DC or because of the secretion of factors that reduce the development and function of DC. When DCs exposed to tumor antigens are instilled, immunity develops. This may result in protection against tumors or a decrease in tumor size, which may represent a potential therapeutic application [50]. DCs are also present in inactivated viruses, which indicates another area for development of vaccines that target them [51].

## 4. Liver Dendritic Cells in Liver Fibrosis

Liver fibrosis and cirrhosis are the end stages of chronic liver disease. Fibrosis is the result of an imbalance between synthesis and degradation of the extracellular matrix (ECM) [52]. The more extensive investigations about the cells contributing to hepatic fibrogenesis have focused on hepatic stellate cells (HSCs) and myofibroblasts (MFs). HSCs possess the capabilities of fibrillar collagen synthesis, chemotactic and vasoactive factor secretion, contractile activity, and production of matrix metalloproteinases (MMPs) and tissue inhibitors of metalloproteinases (TIMPs) [53]. MMPs and TIMPs regulate the turnover of ECM proteins [52]. Soluble factors and different cell types, including Kupffer cells, hepatocytes, and endothelium cells, are able to activate HSCs [54, 55]. Fibrosis is regulated by the adaptive and innate immune systems [52]. Myofibroblasts are the other kind of cells implicated in liver fibrogenesis by producing ECM [56]. Fibrosis is linked to chronic inflammation; therefore, the recruitment of immune cells is one of the hallmarks of fibrogenesis [57]. Recently, Rahman and Aloman proposed a potential mechanism for the contribution of DC to fibrogenesis [11]. They stated that DCs regulate the number and activity of cells involved in the development of fibrosis (such as natural killer cells and CD8^+^ cells), and tissue remodeling, originally attributed to macrophages/monocytes, may be dependent on DC because of their ontogenetic relationship [11].

During the steady state, DCs are immature and tolerogenic. However, in an environment of chronic inflammation, DCs are transformed to a mature proinflammatory subset; interestingly, DCs prevent damage during acute injury [58]. The mature proinflammatory DCs facilitate the deposition of monocytes and activation of HSCs [58]. In addition, in a fibrosis model in mice, it was demonstrated that CD11c^+^ DCs were elevated induced by the administration of thioacetamide (TAA) and recombinant leptin. In this situation, there was an increase in IL-6 and TNF-*α* with the activation of the HSCs [59]. Ibrahim et al. [60] have analyzed the link between lipid metabolism and DC function. The experiments were conducted in NASH mice induced with a methionine/choline-deficient diet, a mice liver fibrosis model induced with TAA, bile duct ligation-induced liver injury in rodents, and human cellular isolation from patients undergoing hepatic resection. Human DCs were lin^−^ HLA-DR^+^, and mice DCs were CD11c^+^ cells. They found two DC populations in both humans and mice defined by high (H-DC) or low (L-DC) lipid content. H-DCs had high concentrations of phospholipids and triglycerides. Cholesterol levels were similar between H-DC and L-DC. The different lipid profiles were associated with the lipid-rich hepatic microenvironment; steatohepatitic human livers showed an increased proportion of H-DC fraction, and mice with NASH also displayed a greater fraction of H-DC. It is noteworthy that H-DCs produced high levels of various proinflammatory CTN and CHM, while L-DCs were virtually nonproductive. The principal CTN found to be elevated were TNF-*α*, IFN-*γ*, IL-6, IL-4, and IL-2. H-DCs also showed an exaggerated response to TLR ligation and a strong T-cell proliferation. Furthermore, H-DC seemed to activate natural killer and natural killer T cells. This evidence suggests that immunogenic DCs are associated with liver lipid content [60], and in NAFLD, this could be an important pathophysiological mechanism with the interplay between the endocrine system and immune cells.

On the other hand, it has been suggested that DC could play a role in the regression of liver fibrosis. Jiao et al. [61] induced liver injury in a murine model using carbon tetrachloride (CCl_4_) and evaluated fibrosis regression after cessation of insult in an environment depleted of DC. In concordance, expansion of DC accelerates hepatic fibrosis regression. Furthermore, Henning et al. [62], using an animal model, found that DCs (CD11c^+^  MHCII^+^ cells) limit CD8^+^ T-cell expansion and restrict TLR expression and cytokine generation from Kupffer cells, neutrophils, and monocytes in NASH. Furthermore, ablation of DC cells resulted in delayed resolution of inflammation and fibroplasia.

There is some evidence that supports the modulation role of DC in liver fibrosis, but it is not clearly understood. Some evidence indicates that DCs promote liver fibrosis, while another study shows otherwise. Indeed, the markers used in the studies conducted so far do not differentiate the DC from other cells such as macrophages or monocytes; therefore, the current understanding about the role of DC is mostly based on the study of MHCII^+^ myeloid or CD11c^+^ cell populations [58]. In the future, efforts will need to concentrate on finding a model capable of differentiating human DC from other myelomonocytic cells to assess their role in liver diseases including NAFLD.

## 5. Conclusions

NAFLD is a complex disease that encompasses a wide range of pathologies, from steatosis to steatosis with inflammation to fibrosis. It will be very important in the near future to know how the inflammatory pathways interact with different cell types and immune tissues resulting in the progression of NASH. In addition, DCs also appear to play an important role in NAFLD; we have shown in this review that a reduction in DC results in a deterioration of NASH severity, suggesting a regulatory role for DC in NASH.

## Figures and Tables

**Table 1:** Different dendritic cell subtypes.

Subset	Phenotype
Lymphoid	CD8*α* ^+^, B220^−^, CD11b^−^, and DX5^−^
Myeloid	CD8*α* ^−^, B220^−^, CD11b^+^, and DX5^−^
Plasmacytoid	CD8*α* ^−^, B220^+^, CD11b^−^, and DX5^−^
Mixed lymphoid + myeloid	CD8*α* ^−^, B220^−^, CD11b^−^, and DX5^−^
NKDC	CD8*α* ^−^, B220^+^, CD11b^lo^, and DX5^+^

NKDC: natural killer dendritic cell.

Modified from [9].

## Glossary

CHM: Chemokines
CTN: Cytokines
ECM: Extracellular matrix
FBR: Fibrogenesis
HIV: Human immunodeficiency virus
HSC: Hepatic stellate cell
IDC: Immature dendritic cell
IL-12: Interleukin-12
DC: Dendritic cells
MDC: Mature dendritic cell
MHC: Major histocompatibility complex
MHCII: Major histocompatibility complex class II
MF: Myofibroblast
MMP: Matrix metalloproteinase
NAFLD: Nonalcoholic fatty liver disease
NASH: Nonalcoholic steatohepatitis
TIMPs: Tissue inhibitors of metalloproteinases
TAA: Administration of thioacetamide
TLR: Toll-like receptor.
